# Influence of adolescents’ tendency to catastrophise on non-suicidal self-injury behaviour: A moderated mediation model

**DOI:** 10.3389/fpsyg.2022.936286

**Published:** 2022-11-16

**Authors:** Zhensong Lan, Kee Pau, Hapsah Md Yusof, Qi Zhao, Fangmei Liang, Xuefang Huang

**Affiliations:** ^1^School of Public Administration, Hechi University, Hechi, China; ^2^National Child Development Research Centre, Sultan Idris Education University, Tanjong Malim, Malaysia; ^3^Department of Psychology and Counseling, Faculty of Human Development, Sultan Idris Education University, Tanjong Malim, Malaysia; ^4^Research and Practice Centre of Rural Basic Education in New Era Minority Region, Hechi University, Hechi, China

**Keywords:** catastrophising tendency, non-suicidal self-injury, NSSI, negative emotions, parental support, adolescents

## Abstract

Adolescence is the peak period of non-suicidal self-injury (NSSI) behaviour, and it is also a period when the prevention and intervention in NSSI are frequently required. To explore the relationship between catastrophising and NSSI and its mechanism, the mediating effects of parental support and negative emotions on catastrophising and NSSI were analysed. A questionnaire was administered to 401 middle school students with a history of NSSI behaviour from 12 schools in Guangxi. (1) Adolescents’ catastrophising tendencies positively predicted their NSSI status through negative emotions (*B* = 0.274, *P* < 0.001). The more severe the adolescents’ catastrophising tendency, the more likely they were to have stronger negative emotions and thus show an increased NSSI level. (2) Parental support played a positive moderating role between the tendency to catastrophise and negative emotions (*B* = −0.101, *t* = −2.694, *P* < 0.01), and it had a protective effect on adolescents’ NSSI. Catastrophising was less likely to induce the NSSI behaviour by reducing adolescents’ negative emotions (*B* = −0.104, *t* = −2.313, *P* < 0.05); however, it should be noted that only parental affective support, informative support, and instrumental support played a significant mediating role. Parental support of adolescents has positive effects on the tendency to catastrophise, negative emotions, and NSSI, and it has some implications for the prevention and intervention of adolescents’ NSSI.

## Introduction

Non-suicidal self-injury (NSSI) is an act where an individual intentionally harms their body without suicidal intent (Hilt et al., 2008). NSSI among adolescents has gradually become a public health problem. Adolescence is the first occurrence and peak period of NSSI in adolescents, which is marked as the beginning of NSSI in individuals (Nock, 2010). NSSI is a potentially lethal risk (Farber et al., 2007), and adolescents are more likely to engage in risky behaviours (Steinberg, 2010). In addition, global data show that NSSI not only endangers the physical and mental health of adolescents, but also leads to suicide (Hawton et al., 2015; Liu et al., 2018; Xu et al., 2019). The incidence of NSSI behaviour among Chinese adolescents ranges from 5.4 to 57.4%, with an average incidence of approximately 29.7% (Zheng, 2006; Feng, 2008; Wan et al., 2011, Zhang et al., 2014; Lin et al., 2018; Tang et al., 2018; Wang et al., 2022). The prevalence of NSSI among adolescents in Western countries ranges from 4 to 56% (Ross and Heath, 2002; Bjärehed and Lundh, 2008; Horváth et al., 2015; Plener et al., 2015), with an average incidence of approximately 27.6% (Plener et al., 2015). This data show that the incidence of NSSI behaviour among Chinese adolescents is prevalent, and they are facing serious problems. There are many factors influencing adolescents’ NSSI behaviour, such as biological factors, cognitive impairment, emotional regulation, and negative life events (Klonsky, 2007; In-Albon et al., 2013; O’Connor and Nock, 2014; Fredlund et al., 2017; Sami and Hallaq, 2018). Research also suggests that brain changes affect patients with early onset mood disorders, even affecting NSSI behaviour (Serafini et al., 2014). Due to the complexity of NSSI behaviour, in 2016, after the promulgation of the Mental Health Law and other laws and policies, 22 departments in China jointly issued the Guiding Opinions on Strengthening Mental Health Services, emphasising the importance of strengthening mental health services and putting forward the requirements of improving China’s mental health service system and improving the mental health literacy of the whole population.

## Theory and hypotheses

Studies have shown that there is a close relationship between emotions and NSSI. Thus, Victor and Klonsky (2014) used diaries to study individuals’ daily emotional experiences, and they found that individuals with NSSI experienced more negative emotions than those without it. Experiential avoidance theory suggests that individuals who adopt NSSI can avoid adverse emotional experiences (Chapman et al., 2006), and individuals with repeated NSSI are more likely to use coping styles to avoid emotions (Nock and Mendes, 2008). Emotional management theory points out that when individuals are affected by strong negative emotions (Chen et al., 2020; Hunnicutt Hollenbaugh et al., 2020), they adopt NSSI to manage such intolerable negative emotions. This is because NSSI is an effective strategy for regulating emotions, which can reduce strong emotional distress or negative emotions (Nock and Prinstein, 2004; Chapman et al., 2006; Klonsky, 2007), and individuals may lack strategies to regulate negative emotions (Xiao et al., 2008) or have difficulties in regulating them (Gratz, 2007), thus causing NSSI. For example, individuals with NSSI have fewer choices to solve problems and tend to use NSSI as an effective coping strategy (Haines and Williams, 2003). In contrast to these two explanations, the theory of psychological interaction emphasises individual differences in the repeated occurrence of NSSI and holds that different individuals have different preferences for rumination. Individuals with a high preference for ruminative thinking will feel excessive psychological pressure when facing stressful events and are more likely to enter a vicious circle of negative experiences, thus aggravating NSSI (Rui et al., 2022).

Catastrophising is one strategy of negative emotion regulation, which refers to an individual’s tendency to magnify the perceived threat and overestimate its potential consequences (Luo et al., 2018). Although the emotional state and emotion regulation ability may have an impact on adolescent NSSI, the way emotions are regulated should be further refined. According to cognitive theory, psychological disorders result from a misunderstanding of environmental events, which directly affect an individual’s mood, behaviour, and physiological state. Studies have shown that catastrophic thinking is the main cause of anxiety, and the more frequently individuals use catastrophising tendency regulation, the more likely they are to suffer from negative emotions, such as anxiety and depression (Gellatly and Beck, 2016). Catastrophising tendencies impact an individual’s emotional state, which is directly related to the individual’s NSSI behaviour. However, previous studies have not explored the relationship between catastrophising tendencies and individual NSSI behaviours. This study attempted to further verify the relationship between catastrophising tendency, emotional state, and NSSI behaviour. **Hypothesis 1:** Adolescent catastrophising tendency is positively correlated with NSSI. **Hypothesis 2:** Adolescents’ negative emotions play a mediating role between the tendency to catastrophise and NSSI.

In addition to the factors of cognitive and emotional regulation in adolescents, attention should also be paid to the impact of the environment on NSSI. The social support theory holds that the social support system experienced by individuals can also have a greater impact on their NSSI (Yang et al., 2015). Furthermore, a good social support system can alleviate an individual’s negative emotional experiences, release negative energy in the heart, and reduce the occurrence of NSSI (Feng, 2017). The family environment also plays a significant role in the growth and development of adolescents, and parents are the “first teachers.” In the multiple social support systems of adolescents, family support is essential in promoting their overall development (Shek and Sun, 2014). It has been found that the parenting style may be another risk factor for adolescent NSSI (Gray et al., 2022). An excessively strict parenting style will affect children’s inferiority and rebellious psychology, including anxiety, depression, and NSSI (Liao et al., 2022). This finding suggests that parenting style may affect the relationship between adolescents’ emotions and NSSI. Further analysis revealed that depression plays a partial mediating role between parental education methods and NSSI (Li et al., 2019). To further explore the influence of parental support on adolescents’ emotional states, this study attempted to analyse the effect of distinct levels of parental support on adolescents’ negative emotions. **Hypothesis 3:** Parental support is negatively correlated with negative emotions and catastrophising. **Hypothesis 4:** Parental support mediates the relationship between catastrophising and negative emotions.

In addition, low family cohesion was found to be a risk factor for NSSI (Cruz et al., 2014; Liang et al., 2014), whereas high family cohesion buffered the impact of adverse factors on individual NSSI (Jiang et al., 2016). Second, higher family functioning can prevent NSSI (Shek and Yu, 2012; Law et al., 2013), and high-quality parental relationships strengthen the role of protecting adolescents from NSSI (Kidd et al., 2006; Taliaferro et al., 2018). In addition, studies have shown that there are gender differences in the incidence of NSSI in adolescents (Wang et al., 2015), and that there are also gender differences when it comes to emotional neglect and NSSI (Gratz et al., 2002). In summary, social support, family environment, family functioning, and parenting style can affect adolescents’ NSSI behaviour. However, research is lacking on the influence of parental support in the family on adolescents’ NSSI behaviour. This study suggests that parental support is a direct factor in family cohesion and functioning, and parental support may also have an impact on adolescents’ NSSI. Therefore, it is worthwhile to further explore whether the level of parental support can improve adolescents’ NSSI behaviour. **Hypothesis 5:** Parental support of adolescents is negatively correlated with NSSI; **Hypothesis 6:** Parental support of adolescents plays a mediating role between catastrophising tendency and NSSI and has a protective effect on adolescents’ NSSI.

In summary, this study mainly explores the relationship and function between adolescent catastrophising tendency and negative emotions, parental support, and NSSI, expanding on the influence mechanism of the tendency to catastrophise on NSSI, which has theoretical and practical significance for preventing and intervening in adolescent mental health problems.

**FIGURE 1:**
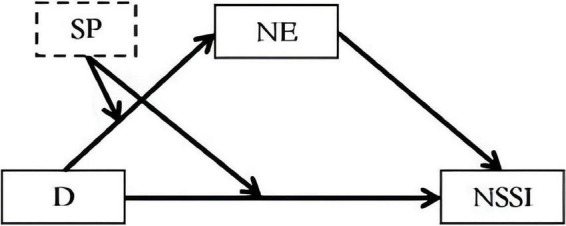
Mediating role of parental emotion and the moderating role of parental support (where D is a catastrophising tendency, SP is parental support, NE is a negative emotion, and NSSI is non-suicidal self-injury).

The research model of this study is presented in Figure 1.

## Materials and methods

### Participants

A total of 2,325 middle school students from 12 ordinary middle schools (including four senior middle schools and eight junior middle schools) in Nanning, Hechi, Yulin, and Qinzhou in Guangxi. The age of the participants ranged from 11 to 18 years, which is the main period of adolescent NSSI behaviour, and is of great significance when studying adolescent NSSI behaviour. In addition, 12 schools were selected for this study which were distributed in different cities in Guangxi, and the sample of adolescents in schools was representative, which can fully reflect the overall characteristics of adolescents in Guangxi, China. In this study, adolescents from 48 classes (two classes in each grade) were selected as the tested samples using the cluster sampling method. Before starting the survey, all participants and their parents were clearly informed of the content and use of the survey data as well as their privacy. After informed consent was obtained from the participants, a formal investigation was conducted. This study was reviewed and approved by the Human Research Ethics Committee of Sultan Idris University of Education, Malaysia (approval number 2022-0465-01).

A total of 2,573 questionnaires were sent out, and 2,400 questionnaires were collected, with a recovery rate of 93.27%. After removing invalid questionnaires, such as consistent responses and multiple missing answers, 2,325 valid questionnaires were obtained, with an overall efficiency rate of 90.36%. To focus on adolescents with NSSI, this study selected 401 adolescents with a history of NSSI behaviour, and the incidence of NSSI was 17.25%. A total of 149 (35.5%) boys and 271 (64.5%) girls experienced NSSI. The average age of the participants was 14.98 ± 1.61 years.

### Research tools

#### Catastrophising tendency scale

The Chinese version of the Cognitive Emotion Regulation Questionnaire (CERQ) revised by Zhu et al. (2007) was tested. The “catastrophising” subscale of CERQ (Zhu et al., 2007) was used as a scale to test the tendency of adolescents to catastrophise. It includes four questions and is scored on a 5-point Likert-type scale. The higher the score, the higher the catastrophic tendency. The Cronbach’s alpha coefficient in this study was 0.88.

#### Emotional self-rating scale

The Simplified Chinese version of the Depression Anxiety Stress Scales (DASS) (Taouk et al., 2001; Gong et al., 2010), which was translated and tested by Gong et al. (2010) according to the traditional Chinese version revised by Taouk et al. (2001), was used. The scale involves three subscales; each subscale contains seven items, for a total of 21 items, and is a 5-point scoring scale; the higher the score, the worse the emotional state of the participants. Cronbach’s alpha in this study was 0.93.

#### Parental support scale

The Chinese version of the Child and Adolescent Social Support Scale revised by Luo et al. (2017) was used. This study mainly used the parental support subscale (i.e., the four dimensions of affective support, instrumental support, evaluative support, and informational support), including 12 questions, using a 6-point scale; the higher the score, the higher the degree of parental support. Cronbach’s alpha in this study was 0.91.

#### Self-rating scale of non-suicidal self-injury

This study used the Chinese version of the Ottawa Self-Injury Inventory (Zhang et al., 2015) revised and tested by Zhang et al. (2015). The questionnaire is a self-rating scale consisting of 28 items, including the frequency of NSSI, location, method, consequences of injury, and seeking treatment. Two-point (yes, no) and 5-point scales were used. Among them, the occurrence frequency of NSSI is “0, no occurrence; 1, at least once; 2, 1–4 times; 3, once a week; 4, almost every day, and the severity of NSSI includes the condition of the injured body part (0, none; 1, at least one part; 2, 2–5 parts; 3, 5–10 parts; 4, 10 or more parts). In this study, Cronbach’s alpha coefficient was 0.89.

### Analytical procedure

The main analysis procedures of this study are as follows:

First, 2,325 target adolescents were analysed using descriptive statistics to understand the incidence of NSSI and gender differences.
Second, the data of 401 adolescents who showed NSSI behaviour were analysed using a structural equation model to test the relationship between negative emotions, catastrophising tendency, and NSSI, and to test the fitting degree of the model.

According to Hayes’s (2012) suggestion, the plug-in PROCESS of SPSS was used to test the mediating effect of parental support and its four sub-dimensions (affective support, informative support, evaluative support, and instrumental support) between catastrophising tendency, negative emotions, and NSSI.

Finally, according to Edwards and Lambert (2007), parental support was divided into high- and low-support levels, and the moderating effects of high- and low-support levels on catastrophising tendency, negative emotions, and NSSI were estimated. In the grouping of parental support level, the high-level group took the data one standard deviation (SD) higher than the mean as the group (M + 1SD), and the low-level group took the data one standard deviation lower than the mean as the group (M–1SD).

## Results

### Common method bias test

Harman’s one-factor test was used to test for common method bias (Luo et al., 2018). The results showed that 18 factors were extracted without a rotation axis, and the variance explained by the first factor was 16.49% (< 40%), indicating that there was no serious common method bias in our data.

### Descriptive statistics

There was no significant difference in gender (*F* = 0.18, *t* = 0.06, *P* > 0.05) and age [*F*(6) = 0.62, *P* > 0.05] among adolescents who experienced NSSI. The means, SDs, and correlation matrices for each variable are presented in Table 1.

**TABLE 1:** Descriptive statistics (*N* = 401).

Variables	*M*	*SD*	1	2	3	4	5	6	7	8
1. NSSI	9.89	7.75	1							
2. Catastrophe tendency	1.60	1.14	0.240***	1						
3. Negative emotions	32.58	17.06	0.315***	0.594***	1					
4. Parental support	26.89	11.70	−0.170***	−0.249***	−0.279***	1				
5. Emotional support	2.26	1.09	−0.159***	−0.273***	−0.317***	0.854***	1			
6. Informational support	2.43	1.26	−0.130**	0.169*	−0.228***	0.853***	0.650***	1		
7. Evaluative support	2.20	1.24	−0.141**	−0.227***	−0.249***	0.845***	0.646***	0.596***	1	
8. Instrumental support	2.07	1.05	−0.143**	−0.170***	−0.140**	0.805***	0.586***	0.588***	0.570***	1

*P < 0.05, **P < 0.01, ***P < 0.001.

The results show that the tendency to catastrophise was positively correlated with negative emotions (*t* = 0.594, *P* < 0.001) and NSSI (*t* = 0.240, *P* < 0.001); parental support was negatively correlated with catastrophising (*t* = −0.249, *P* < 0.001), negative emotions (*t* = −0.279, *P* < 0.001), and NSSI (*t* = −0.170, *P* < 0.01). In addition, among the four sub-dimensions of parental support, affective, informational, evaluative, and instrumental support were significantly negatively correlated with catastrophic self-evaluation, negative emotions, and NSSI. Therefore, Assumptions 1, 3, and 5 are true.

### Relationship between catastrophising tendency and non-suicidal self-injury: Moderated mediating effect test

#### Testing the mediation effect in structural equation modelling

**FIGURE 2:**
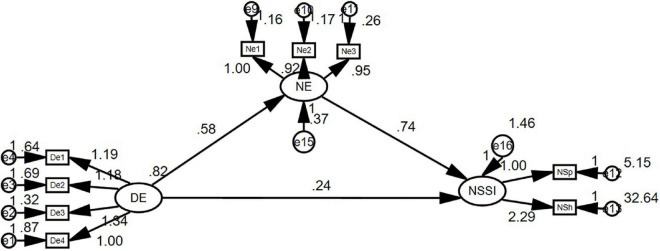
Structural equation model fit test (*N* = 401, where DE is catastrophising tendency, NE is a negative emotion, and NSSI is non-suicidal self-injury).

Zhao et al. (2010) argued that the SEM approach is the best framework for analysing mediating effects (Iacobucci et al., 2007). The bootstrapping method was used to generate the empirical sample distribution of the statistic (mediating effect), from which the confidence interval (CI) and standard error were obtained to determine the statistical significance of the mediating effect (Zhao et al., 2010). To confirm and compare the mediating role of negative emotions among catastrophising tendency, parental support, and NSSI, and the mediating role of parental support among catastrophising tendency, negative emotions, and NSSI, AMOS23.0 was used to run the bootstrap method, which was repeated 5,000 times with a 95% CI. The bias correction method was used to test the degree of fit of the constructed model, and the results showed that (see Figure 2), the fitting index of the model was χ^2^/DF = 1.776, GFI = 0.977, AGFI = 0.957, NFI = 0.977, TLI = 0.985, CFI = 0.990, and RMSEA = 0.044. The above fitting indices are in line with the fitting standard, indicating that the fitting effect of the model is better.

In structural equation modelling, negative emotion was used as a mediator to test the mediating effect between catastrophising tendencies and NSSI. The standardised mediating effects of the above variables were calculated by assigning all relevant paths through self-written grammar. In the mediation path of catastrophising tendency → negative emotion → NSSI, the standardised coefficient of the prediction effect of catastrophising tendency on NSSI was *B* = 0.152, *P* < 0.001, and the standardised coefficient of the mediation prediction effect of negative emotion between catastrophising tendency and NSSI was *B* = 0.274, *P* < 0.001, 95% CI was (0.1171, 0.4566) (excluding 0), indicating that negative emotion has a significant mediating effect between catastrophising tendency and NSSI, and the mediating effect is 0.274/(0.274 + 0.152), that is, 64.32%, so Hypothesis 2 was established.

#### Testing the mediating and moderating effect of parental support on catastrophising tendency, negative emotion, and non-suicidal self-injury

To examine the mediating and moderating effect of parental support on catastrophising, negative emotions, and NSSI, this study tested Hypothesis 4 by using PROCESS Model 8 from SPSS macros compiled by Hayes (2012).

When gender, ethnicity, and age were controlled, catastrophising tendency positively predicted negative emotion (*B* = 0.542, *t* = 13.106, *P* < 0.001), while parental support negatively predicted negative emotion (*B* = −0.142, *t* = −3.468, *P* < 0.001). Catastrophising tendency was used as the independent variable, parental support as the moderator variable, and negative emotion as the dependent variable. When parental support was added to the model, the product of catastrophising tendency and parental support was a significant predictor of negative emotions (*B* = −0.101, *t* = −2.694, *P* < 0.01).

When controlling for gender, ethnicity, and age, and taking catastrophising as the independent variable, parental support as the moderator, and NSSI as the dependent variable, catastrophising did not positively predict NSSI (*B* = 0.080, *t* = 1.360, *P* > 0.05), but emotional support (*B* = 0.234, *P* > 0.05). Parental support was not a significant negative predictor of NSSI (*B* = −0.077, *t* = −1.559, *P* > 0.05), but the product of catastrophising and parental support was a significant predictor of NSSI (*B* = −0.104, *t* = −2.313, *P* < 0.05).

These results suggest that parental support has a significant moderating effect between catastrophising and negative emotions and that parental support had a significant moderating effect between catastrophising, negative emotions, and NSSI. The above analysis results are shown in Table 2. Therefore, Assumption 4 holds.

**TABLE 2:** Moderated mediation model test of parental support (*N* = 401).

Result variable	Negative emotion			NSSI		
Predictor variable	*B*	*SE*	*t*	*B*	*SE*	*t*
Gender	0.051	0.040	1.278	–0.212	0.048	–0.458
Nation	0.003	0.040	0.070	–0.107	0.048	−2.233*
Age	0.049	0.040	1.236	0.018	0.047	0.385
Catastrophe tendency	0.542	0.041	13.106***	0.080	0.059	1.360
Negative emotion				0.234	0.060	3.896***
Parental support	–0.142	0.041	−3.468***	–0.077	0.050	–1.559
Catastrophe tendency × parental support	–0.101	0.038	−2.694**	–0.104	0.045	−2.313*
*R*		0.622			0.367	
*R* ^2^		0.387			0.135	
*F*		41.432***			8.732***	

*P < 0.05, **P < 0.01, ***P < 0.001.

**FIGURE 3:**
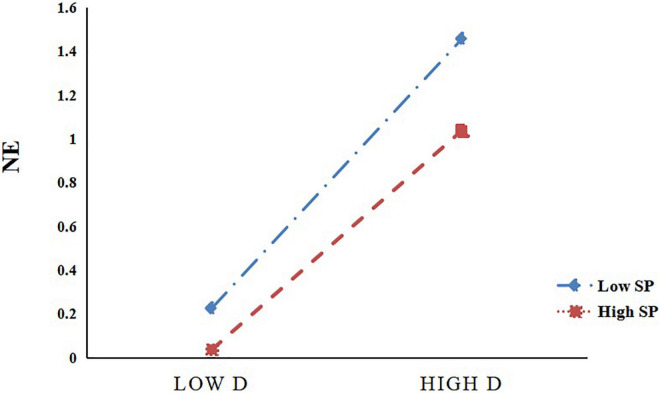
Parental support as a moderator between catastrophising and negative emotions (*N* = 401, where D is catastrophising, SP is parental support, NE is negative emotions, and NSSI is non-suicidal self-injury).

It can be seen from Figure 3 that for the participants with low parental support (M–1SD), the catastrophising tendency had a significant positive predictive effect on negative emotions, *B* = 0.644, *t* = 12.306, *P* < 0.001, and the 95% CI was (0.5408, 0.7464). For participants with higher levels of parental support (M + 1SD), the tendency to catastrophise had a positive predictive effect on negative emotions, but its predictive effect was smaller [*B* = 0.441, *t* = 7.439, *P* < 0.001, 95% CI (0.3245, 0.5577)]. The above results show that with an increase in parental support, the predictive effect of the tendency to catastrophise on negative emotions gradually decreases.

**FIGURE 4:**
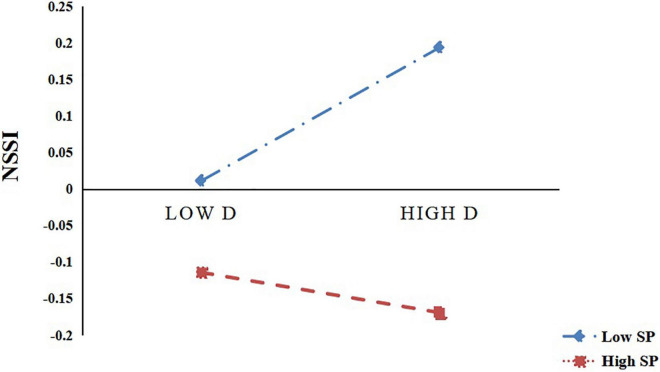
Parental support as a moderator between catastrophising and NSSI (*N* = 401, where D is catastrophising, SP is parental support, NE is negative affect, and NSSI is non-suicidal self-injury).

It can be seen from Figure 4 that the tendency to catastrophise had a significant positive predictive effect on NSSI in participants with low parental support (M–1SD), *B* = 0.185, *t* = 2.522, *P* < 0.05, and the 95% CI was (0.0407, 0.3285); for the participants with higher levels of parental support (M + 1SD), the predictive effect of catastrophising tendency on NSSI was not significant, *B* = −0.024, *t* = −0.320, *P* > 0.05, and the 95% CI was -0.1722, 0.1240. The above results indicate that with an increase in parental support, the predictive effect of catastrophising on NSSI gradually decreases. Hypothesis 6 was tested using Edwards and Lambert’s (2007) proposal. At the three levels of parental support, the mediating effect of negative emotions on the relationship between catastrophising tendency and NSSI also showed a decreasing trend (see Table 3); that is, with an increase in the level of parental support, catastrophising tendency was less likely to induce adolescents’ NSSI behaviour by reducing their negative emotions. Hence, Hypothesis 6 holds.

**TABLE 3:** Mediating effects at different levels of parental support (*N* = 401).

Type	Indicator	Effect value	Boot S.E.	Boot CI lower	Boot CI upper
Mediated mediating effect	−1.0 (M–1SD)	0.150	0.045	0.068	0.245
	0 (M)	0.127	0.039	0.056	0.209
	1.0 (M + 1SD)	0.103	0.034	0.044	0.177

#### The four sub-dimensions of parental support mediate the relationship between catastrophising tendency, negative emotion, and non-suicidal self-injury

To examine the mediating effect of different levels of parental support on the tendency to catastrophise, negative emotions, and NSSI in adolescents, similarly, when controlling for gender, ethnicity, and age, the PROCESS Model 8 in the SPSS macro compiled by Hayes (2012) was still used to test the mediating and moderating effect of these four sub-dimensions. The results are presented in Table 4.

**TABLE 4:** Mediation model test of parental support on four sub-dimensions (*N* = 401).

	Result variable	Negative emotion			NSSI		
	Predictor variable	*B*	*SE*	*t*	*B*	*SE*	*t*
Affective support dimension	Constant 1	–0.026	0.041	–0.639	–0.031	0.049	–0.642
	Catastrophe tendency	0.530	0.042	12.759***	0.083	0.059	1.397
	Affective support	–0.180	0.041	−4.354***	–0.056	0.051	–1.101
	Negative emotion				0.238	0.060	3.938***
	Catastrophe tendency × affective support	–0.095	0.038	−2.493*	–0.114	0.046	−2.487*
Informative support dimension	Constant 2	–0.014	0.040	–0.336	–0.014	0.048	–0.282
	Catastrophe tendency	0.065	0.041	13.905***	0.092	0.059	1.557
	Informative support	–0.118	0.041	−2.885**	–0.045	0.049	–0.921
	Negative emotion				0.246	0.060	4.106***
	Catastrophe tendency × informative Support	–0.080	0.038	−2.133*	–0.080	0.045	–1.783
Evaluative support dimension	Constant 3	–0.015	0.041	–0.358	–0.010	0.048	–0.198
	Catastrophe tendency	0.555	0.042	13.384***	0.084	0.059	1.413
	Evaluative support	–0.125	0.041	−3.058**	–0.059	0.049	1.198
	Negative emotion				0.251	0.060	4.201***
	Catastrophe tendency × evaluative support	–0.064	0.039	1.671	–0.042	0.046	–0.921
Instrumental support dimension	Constant 4	–0.017	0.040	–0.409	–0.019	0.047	–0.397
	Catastrophe tendency	0.575	0.041	14.003***	0.082	0.059	1.388
	Instrumental support	–0.038	0.0441	–0.930	–0.085	0.048	–1.774
	Negative emotion				0.242	0.590	4.099***
	Catastrophe tendency × instrumental support	–0.098	0.037	−2.621**	–0.111	0.044	−2.526*

*P < 0.05, **P < 0.01, ***P < 0.001.

Taking the tendency to catastrophise as the independent variable, parental affective support as the moderator, and negative emotion as the dependent variable, the product of the tendency and parental affective support had a significant predictive effect on negative emotions (*B* = −0.095, *t* = −2.493, *P* < 0.05). Taking the tendency to catastrophise as the independent variable, parental effective support as the moderator variable, and NSSI as the dependent variable, the product of the tendency and parental affective support had a significant predictive effect on NSSI (*B* = −0.114, *t* = −2.487, *P* < 0.05). According to the results in Table 5, it can be seen that parents’ affective support plays a mediating and moderating role in “Catastrophe tendency→Negative emotion→NSSI,” and the Moderator value is -0.315.

**TABLE 5:** Index of parental support’s moderated mediation on four sub-dimensions (*N* = 401).

Path	Index	Boot S.E.	Boot CI lower	Boot CI upper	J.N. value
M1	–0.023	0.011	–0.0477	–0.0040	–0.315
M2	–0.020	0.011	–0.0416	–0.0001	–0.357
M3	–0.016	0.010	–0.0380	0.0019	
M4	–0.023	0.011	–0.0474	–0.0060	–0.338

M1, M2, M3, and M4 represent the mediating roles of affective support, informative support, evaluative support, and instrumental support in the “Catastrophe tendency→Negative emotion→NSSI” pathway, respectively; J.N. value is the moderator value(s) defining Johnson-Neyman significance region(s).

Taking the tendency to catastrophise as an independent variable, parental informative support as the moderator variable, and negative emotion as the dependent variable, the product of the tendency and parental informative support had a significant predictive effect on negative emotions (*B* = −0.080, *t* = −2.133, *P* < 0.05). Taking the tendency to catastrophise as an independent variable, parental informative support as the moderator, and NSSI as the dependent variable, the product of catastrophising and parental informative support had no significant predictive effect on NSSI (*B* = −0.080, *t* = −1.783, *P* > 0.05). According to the results in Table 5, it can be seen that parents’ informative support plays a mediating and moderating role in “Catastrophe tendency→Negative emotion→NSSI,” and the Moderator value is -0.357.

When the tendency to catastrophise was taken as the independent variable, parental evaluative support as the moderator, and negative emotion as the dependent variable, the product of the tendency and parental evaluative support had no significant predictive effect on negative emotion (*B* = −0.064, *t* = 1.671, *P* > 0.05). Taking the tendency to catastrophise as an independent variable, parental evaluative support as the moderator, and NSSI as the dependent variable, the product of catastrophising and parental evaluative support had no significant predictive effect on NSSI (*B* = −0.042, *t* = −0.921, *P* > 0.05). According to the results in Table 5, it can be seen that parents’ evaluative support plays no mediating and moderating role in “Catastrophe tendency→Negative emotion→NSSI.”

With the tendency to catastrophise as an independent variable, parental instrumental support as the moderator variable, and negative emotion as the dependent variable, the product of the tendency and parental instrumental support had a significant predictive effect on negative emotions (*B* = −0.098, *t* = −2.621, *P* < 0.01). Taking the tendency to catastrophise as an independent variable, parental instrumental support as the moderator, and NSSI as the dependent variable, the product of the tendency and parental instrumental support had a significant predictive effect on NSSI (*B* = −0.111, *t* = −2.526, *P* < 0.05). According to the results in Table 5, it can be seen that parents’ instrumental support plays a mediating and moderating role in “Catastrophe tendency→Negative emotion→NSSI,” and the Moderator value is -0.338.

These results suggest that the affective, informative, and instrumental support of parents plays a significant role in mediating and moderating the relationship between the catastrophising tendency, negative emotions, and NSSI. However, parents’ evaluative support did not play a significant role in mediating and moderating the relationship between catastrophising tendency, negative emotions, and NSSI.

## Discussion

This study found that the emotional state of adolescents was positively correlated with NSSI behaviour, verifying the correlation between emotional state and NSSI behaviour. Although the data of this study cannot effectively explain the causal relationship between negative emotions and NSSI behaviour, these data partially support the experiential avoidance theory and emotional management theory to explain the relationship between negative emotions and NSSI behaviour. This study also verified the correlation between adolescents’ catastrophising tendency and emotional state, and supported the conclusion that catastrophising tendency can lead to individual negative emotions (Gellatly and Beck, 2016). In addition, this study also found that negative emotions play a significant mediating role in the prediction of adolescents’ NSSI by catastrophising tendency; that is to say, adolescents with catastrophising tendencies may increase their negative emotions, thereby increasing the risk of NSSI.

According to the social support theory, social support systems may have a greater impact on individual NSSI (Yang et al., 2015), and social support systems can alleviate individual negative emotional experiences and reduce the occurrence of NSSI (Feng, 2017). The results of this study show that parental support plays a mediating role in the relationship between negative emotions and NSSI, and that adolescents with high levels of parental support are less affected by negative emotions to produce NSSI, which indicates that parental support buffers the relationship between negative emotions and NSSI. However, it is worth noting that only affective, informative, and instrumental support played a significant mediating role in this relationship. Therefore, parents’ emotional, informational, and instrumental support are effective for adolescents who display NSSI behaviour, and they expect to receive more support from their parents in terms of emotional warmth, information, and material satisfaction. These adolescents were not interested in their parents’ evaluative support and refused to be lectured, criticised, or advised by their parents.

This study also found that adolescents with higher levels of parental support were less likely to engage in catastrophising, thinking about what they were facing, and avoiding negative emotional reactions. This positive attitude weakens the relationship between catastrophising tendencies and NSSI, verifies the role of family support in promoting the overall development of adolescents (Shek and Sun, 2014), and helps to protect adolescents from NSSI (Kidd et al., 2006; Liu et al., 2018; Taliaferro et al., 2018). Therefore, providing parental support can bring hope to adolescents affected by NSSI.

This study expands the research on the influence mechanism of the tendency to catastrophise on NSSI among adolescents and has certain educational and practical implications for the prevention and intervention of adolescent NSSI. On the one hand, people can predict adolescents’ NSSI behaviour through a negative emotional state; In contrast, by providing appropriate parental support, we can reduce the negative effect of catastrophising tendencies on NSSI behaviour.

The specific measures are as follows: First, it is necessary to improve the self-cognition levels of adolescents and cultivate awareness of facing their negative emotions; in particular, psychological education should be strengthened for adolescents to learn emotional regulation skills to cope with pressure correctly, to reduce the tendency of catastrophising, and prevent and reduce the negative influence of NSSI behaviour. Second, attention should be paid to the influence of family environment and parental support on adolescent NSSI. The relationship between adolescents and their parents can be established and strengthened to improve the level of parental support, thereby buffering the impact of youth catastrophising tendency on negative emotions and NSSI. Finally, we should strengthen the relationship between schools and parents, play the role of parental support, and provide adequate services for adolescents with NSSI. In conclusion, attention should be paid to the negative effects of catastrophising tendencies on adolescents and the positive effects of parental support when designing and developing service strategies or courses for adolescent NSSI behaviour, which is helpful in improving NSSI behaviour service strategies for adolescents.

This study has several limitations. Firstly, in terms of sample selection, all the participants were from five cities in Guangxi; the total number of samples was not high and the representativeness was limited; and there may be differences in the characteristics and status of different types of adolescents in different regions; therefore, it is necessary to expand the diversity of national samples in the future to test whether the research conclusions are universally applicable. Secondly, although this study tested the mediation model between catastrophising tendency and adolescents’ NSSI and also verified the influence of parental support on adolescents’ negative emotions and NSSI, it could not provide more rigorous and reasonable evidence of a causal chain. That is to say, the path of catastrophising tendency from parental support, negative emotions to NSSI has not been supported by effective evidence, and although catastrophising tendency has a significant positive predictive relationship with NSSI, its role in the chain mediation model of multiple variables has not been explored further; thus, more variables need to be included to improve the fitting degree of the model. Moreover, as the study was a cross-sectional study, there may be some differences in the NSSI of adolescents in different periods and stages. The mediating effect of parental support on catastrophising tendency and negative emotions, as well as the mediating effect of parental support on catastrophising tendency and NSSI, have only been theoretically verified. In the future, we can also explore the mediating role of negative emotions through a longitudinal tracking design, as well as the moderating role of parental support through an experimental design.

## Conclusion

The study conclusions are as follows: (1) There is a significant positive correlation between adolescents’ tendency to catastrophise and negative emotions and NSSI; this tendency positively predicts the NSSI status through negative emotions; when adolescents are faced with a variety of learning, interpersonal, and life pressures, the more serious the catastrophic tendency, the more likely it is to produce strong negative emotions, thereby increasing the NSSI level. (2) Parental support was negatively correlated with catastrophising, negative emotions, and NSSI; parental support plays a positive regulatory role between the tendency to catastrophise and negative emotions, and it has a protective effect on adolescents’ NSSI; that is, with the improvement of parental support, it is more difficult for catastrophising tendency to induce adolescents’ NSSI behaviour by reducing their negative emotions. However, only parental affective, informative, and instrumental support played a significant mediating role in this relationship.

## Data availability statement

The original contributions presented in this study are included in the article/supplementary material, further inquiries can be directed to the corresponding author.

## Ethics statement

The studies involving human participants were reviewed and approved by the Human Research Ethics Committee of Sultan Idris University of Education, Malaysia (approval number: 2022-0465-01). Written informed consent to participate in this study was provided by the participants’ legal guardian/next of kin.

## Author contributions

KP and HY focused on the conception and design of the study. ZL, QZ, and XH focused on the acquisition of data, drafting the article, and revising it critically for important intellectual content. ZL and FL focused on the analysis and interpretation of data. All authors contributed to the article and approved the submitted version.
